# Computational Complexity and Its Influence on Predictive Capabilities of Machine Learning Models for Concrete Mix Design

**DOI:** 10.3390/ma16175956

**Published:** 2023-08-30

**Authors:** Patryk Ziolkowski

**Affiliations:** Faculty of Civil and Environmental Engineering, Gdansk University of Technology, Gabriela Narutowicza 11/12, 80-233 Gdansk, Poland; patziolk@pg.edu.pl; Tel.: +48-58-347-2385

**Keywords:** applied machine learning, buildings, cement, concrete mix design, concrete strength prediction, concrete, construction industry, data mining, green building, innovation, sustainability, sustainable development, sustainable

## Abstract

The design of concrete mixtures is crucial in concrete technology, aiming to produce concrete that meets specific quality and performance criteria. Modern standards require not only strength but also eco-friendliness and production efficiency. Based on the Three Equation Method, conventional mix design methods involve analytical and laboratory procedures but are insufficient for contemporary concrete technology, leading to overengineering and difficulty predicting concrete properties. Machine learning-based methods offer a solution, as they have proven effective in predicting concrete compressive strength for concrete mix design. This paper scrutinises the association between the computational complexity of machine learning models and their proficiency in predicting the compressive strength of concrete. This study evaluates five deep neural network models of varying computational complexity in three series. Each model is trained and tested in three series with a vast database of concrete mix recipes and associated destructive tests. The findings suggest a positive correlation between increased computational complexity and the model’s predictive ability. This correlation is evidenced by an increment in the coefficient of determination (R^2^) and a decrease in error metrics (mean squared error, Minkowski error, normalized squared error, root mean squared error, and sum squared error) as the complexity of the model increases. The research findings provide valuable insights for increasing the performance of concrete technical feature prediction models while acknowledging this study’s limitations and suggesting potential future research directions. This research paves the way for further refinement of AI-driven methods in concrete mix design, enhancing the efficiency and precision of the concrete mix design process.

## 1. Introduction

The field of concrete mix design has seen significant advancements in recent years with the integration of machine learning techniques. The ability of these models to predict the compressive strength of concrete has the potential to revolutionise the construction industry by providing more efficient and accurate methods for mix design. However, the prediction accuracy of these models depends on many factors, including the computational complexity of the model itself. This study aims to investigate the influence of computational complexity on the prediction accuracy of concrete compressive strength in machine learning models for concrete mix design. The findings of this research will provide insights into the trade-off between model accuracy and computational efficiency and will guide the development of more effective machine learning models for concrete mix design in the future. The concrete mix composition consists of cement, water, a combination of fine and coarse aggregates, and supplementary materials referred to as additives and admixtures, where additives are incorporated during the cement manufacturing stage, whereas admixtures are introduced during concrete mix preparation. These substances are formulated to enhance the chemical properties and performance of concrete, specifically with regard to compressive strength, durability, and workability. There are various types of additives and admixtures [1,2,3,4], including accelerators, substances that improve fresh concrete properties [5], materials that enhance durability, fibres that reinforce concrete [6], set-retarding admixtures, and water-reducing agents.

Properly designing a concrete mixture is a crucial aspect of the construction process, with multiple factors to consider. It should be designed with economy in mind, ensuring that the desired properties can be achieved using the most cost-effective raw materials. The mixture must also be optimised for the specific technology used in the construction process, considering elements such as workability and setting speed. Environmental conditions, such as temperature [7,8], precipitation, distance away from the construction area, as well as the volume of traffic, must also be considered when designing the concrete mix. The final composition of the mix is determined by construction specifications, such as the desired compressive strength or resistance to environmental elements such as chloride ingression and, increasingly, ecological considerations, such as low emissivity. To address environmental concerns, various solutions exist to reduce concrete carbonation, including admixtures of graphene nanoparticles. Designing a concrete mix involves selecting the proper proportions of primary and secondary components to achieve the desired properties. Once the mix is prepared, it is conveyed to the construction location and poured into the formwork, where it undergoes the progression of hardening and increasing strength. The hardening process is initiated by the cement’s hydration, which involves a heat-releasing chemical reaction that occurs upon contact between cement and water [9]. The reaction initiates the formation of various components, such as tobermorite gel [10], hydroxide, as well as additional components, which improve the bonding between coarse and fine aggregates. During this procedure, the hydration products steadily accumulate on the cement grains and replace the water in the mixture. The ultimate hydration stage occurs when all the water molecules are fully integrated, or no unreacted cement is left in the mixture. After the hydration process begins, hardened concrete acquires some of its compressive strength within a few days, and most of its compressive strength is attained after roughly 28 days (although some types of concrete may take longer to reach their full strength). The quantity of water required to hydrate the cement completely ranges from 20% to 25% of its weight, excluding water trapped in pores. However, specific models suggest that 42% of the cement’s weight is needed for proper hydration [11]. The design methods for concrete mixtures currently employed in engineering practice have been derived from solutions developed more than a decade ago and rely on estimating the bending strength of the concrete mortar. Implementing these techniques in practice might be a tedious and inefficient process that does not consider the intricate chemical composition and variability of modern concrete mixes. The current challenges in the field at hand necessitate novel technological solutions. Machine learning-based methods could offer a promising avenue, as they have demonstrated varying degrees of success in predicting concrete compressive strength.

Machine learning, a prominent subfield of artificial intelligence, has garnered significant attention in recent years due to its vast applications and transformative potential across various domains. The fundamental concept behind machine learning involves empowering computers to acquire knowledge from data, recognize patterns, and arrive at informed choices while minimizing the need for extensive human involvement. By utilising algorithms and statistical models, machine learning systems can adapt and improve their performance over time, making them valuable tools for a multitude of tasks, ranging from natural language processing [12] and image recognition [13] to real estate value forecasting [14,15,16,17] and medical purposes [18]. The foundation of machine learning lies in its ability to extract knowledge from data, which is achieved by employing various learning paradigms. These paradigms include supervised, unsupervised, and reinforcement learning, each catering to different problem domains. Supervised learning, the most common approach, involves training a model using labelled data with known outcomes, while unsupervised learning deals with discovering hidden structures in unlabelled data [19]. Reinforcement learning, on the other hand, focuses on learning through trial and error, with a model receiving feedback in the form of rewards or penalties [20,21]. A plethora of algorithms have been developed for each learning paradigm, and the choice of the algorithm largely depends on the specific problem and the available data. Popular algorithms include linear regression, decision trees, support vector machines, neural networks, and clustering algorithms. These algorithms often involve tuning various parameters, known as hyperparameters, to optimise the model’s performance. Machine learning has proven particularly effective in addressing complex problems with high-dimensional data. This has been facilitated by the advent of deep learning, a subset of machine learning that relies on artificial neural networks with multiple layers. These networks, drawing inspiration from the organization and operation of the human brain, excel in acquiring complex patterns and representations from extensive datasets, making them suitable for a diverse array of uses, including natural language processing and computer vision. The success of machine learning in diverse fields has prompted researchers to investigate its potential for predicting and optimising properties of materials, such as concrete.

In this paper, the influence of computational complexity on the performance of machine learning models used for predicting the compressive strength of concrete is explored. This research assessed three sets of five deep neural network models (MLM1, MLM2, MLM3, MLM4, MLM5), each with differing levels of computational complexity. Through an examination of various machine learning models, the goal is to identify the trade-offs between accuracy and computational efficiency. This could provide valuable insights into the development of robust and cost-effective models for concrete mix design.

## 2. Concrete Mix Design and Machine Learning

### 2.1. Prediction of Concrete Technical Properties in Concrete Mix Design

Designing an optimal concrete mix is a multifaceted challenge requiring a comprehensive understanding of concrete technology and significant practical experience. The primary objective of the design process is to determine suitable material compositions to achieve the desired properties in fresh concrete during transportation and placement and in hardened concrete. Distinct mechanical properties are anticipated at each stage of the concrete fabrication process. Various characteristics influence concrete performance, including plasticity, durability, compressive strength, and modulus of elasticity. The significance of these properties may vary at different stages, for instance, adequate compressive strength is crucial for the designed ultimate limit state, whereas sufficient durability is critical in aggressive environments. Designing a mix with inappropriate specifications can result in severe consequences. Therefore, fearing noncompliance with the necessary criteria, concrete mix manufacturers often intentionally exceed the designed parameters [22].

Global corporate engineering practices related to concrete technology exhibit considerable variation and notable commonalities. Within the European Union, the primary standard governing concrete technology issues is EN 206 Concrete: Specification, Performance, Production, and Conformity [23], while EN 1992-1-1: Eurocode 2: Design of Concrete Structures [24] provides guidelines for the design of concrete structures. Both standards have national equivalents and appendices, such as DIN EN 206 [25] in Germany and PN-EN 206 + A1: 2016-12 in Poland. Member states of the European Union employ diverse methods for designing concrete mixes. In Poland, the Bukowski, Eyman, Klaus, Kopycinski, and Paszkowski methods are predominantly used alongside the double-coating method. Conversely, the Bolomey, Fuller, and 0.45 power gradation chart methods are more prevalent in the United States. Most of these approaches are derived from the “three equations method” representing a combined experimental-analytical strategy for concrete mix design.

The approach that combines experimental and analytical methods involves determining the required quantity of ingredients through analytical calculations and confirming its accuracy through destructive lab testing. This technique enables the researchers to establish the proportions of cement, water, and aggregate by weight for a given volume, employing three formulas related to consistency (1), strength (2), and impermeability (3). The consistency formula (1) is integrated into the water requirement equation, which assists in identifying the optimal consistency. The demand for water in cement and aggregate relies on factors such as particle size, form, texture, ratio within the mixture, and the desired consistency of the concrete blend. To account for the water needs of concrete additives and admixtures, they are added to the aggregate or cement based on the particle dimensions. Factors such as grain size, shape, surface roughness, composition proportion, and the concrete mix’s required consistency influence the cement–water and aggregate–water demand indices created by Bolomey and Stern [26].

(1) $$W=C\cdot w_{c}+K\cdot w_{k},$$

(2) $$f_{cm}=A\left[\left(\frac{C}{W}+p\right)-a\right],$$

(3) $$f_{cm}=A_{1,2}\left(\frac{C}{W}\pm a\right),$$

(4) $$\frac{C}{\rho_{c}}+\frac{K}{\rho_{k}}+W=1000,$$

In Equation (1), W denotes the volume of water (in litres) present in one cubic meter of concrete. The weight of cement in one cubic meter of concrete is represented by C (in kilograms). The cement–water demand index $w_{c}$, indicates the volume of water (in litres per kilogram) required to be combined with one kilogram of a specific cement class in one decimetre cubed. K represents aggregate weight in one cubic meter of concrete, measured in kilograms. Lastly, the aggregate–water demand index $w_{k}$, signifies the volume of water (in litres per kilogram) needed to be added to one kilogram of a certain dry aggregate fraction to achieve the desired consistency. The subsequent formula, called the concrete compressive strength formula, exists in two variations: Bolomey and Feret. This formula illustrates the connection between concrete compressive strength and factors such as water–cement ratio, cement grade, and aggregate grade. The Feret and Bolomey versions of the formula are represented by Equations (2) and (3), respectively. In these equations, $f_{cm}$ denotes the average concrete compressive strength in MPa, while A, $A_{1}$, and $A_{2}$ are coefficients contingent upon the aggregate’s type and strength class and the cement’s strength class. $A_{1}$ is utilised when the cement-to-water ratio (C/W) is less than 2.5, and $A_{2}$ is employed when the ratio exceeds 2.5. In the equations, C signifies the cement weight in 1 cubic meter of concrete (measured in kg), *W* represents the water quantity in 1 cubic meter of concrete (measured in L), *p* is the air content in one cubic meter of concrete (measured in dm^3^), and a is a numeric value that depends on cement and aggregate quality, typically considered constant equal to 0.5. The a value is positive for water–cement ratios greater than or equal to 2.5, while it is negative for ratios below 2.5. The Feret equation is applicable when the aggregate strength is inferior to the grout strength, specifically in the case of porous concrete. The water-tightness equation, designated as Equation (4), asserts that the total volume of individual components in a concrete mix is equivalent to the mix’s overall volume. In this equation, W signifies the water content in litres per cubic meter (m^3^) of concrete, while C stands for the cement weight in kilograms per cubic meter (m^3^) of concrete. Additionally, $\rho_{c}$ represents the density of cement in kg/dm^3^, K denotes the weight of cement in kg per cubic meter (m^3^) of concrete, and $\rho_{k}$ refers to the aggregate density in kg/dm^3^. Using the equations mentioned above, one can determine the quantitative composition of a concrete mix, which consists of the quantities of cement, water, and aggregate in a cubic meter (m^3^) of the mixture. The three equations method does have specific boundary constraints, for instance, the porosity of the concrete mix must not surpass 0.002 of the mix volume without air-entraining additives or 0.008 of the mix volume when incorporating air-entraining additives.

The process of creating a concrete mix design incorporates several steps such as formulating initial suppositions, defining the necessary characteristics of both the fresh and cured concrete, selecting and assessing the components of the mix, crafting the blend, testing its properties in a lab setting, and finally devising a workable formula. In formulating initial suppositions, key considerations include the concrete’s intended application and the specific traits of the new structure. These factors include location, degree of reinforcement, and the structural cross-section’s geometric properties. The primary attributes of interest for fresh concrete are its bulk density, consistency, and air content. As for the hardened concrete, we look at its frost and fire resistance and the grade of its compressive strength. It is essential to scrutinise the technological process and evaluate the conditions under which the concrete matures and the method used for compacting the fresh mix. The concrete exposure class is a significant parameter, defining the level and nature of environmental stress the material can withstand. Further specifications such as the concrete’s impermeability need to be established. Parameters such as maximum aggregate size and mix workability also need determination. The components of the concrete mix, including the appropriate type of cement, water, and aggregate quality, should be selected and appraised as per relevant standards. Following the mix design and lab tests, the final stage involves creating a functional formula for one cubic meter of the concrete mix. One should also anticipate potential recipe modifications due to the aggregate’s moisture content and adjust it according to specific circumstances, such as the transportation vehicle’s capacity [27,28].

### 2.2. Machine Learning in Prediction of Concrete Technical Properties

Machine learning has permeated numerous scientific domains, showcasing its versatility and potency. It has a particularly large number of applications in the field of structural and material engineering. Machine learning has found use within this branch of science in areas such as structural health monitoring, crack detection, life-cycle cost assessment, or prediction of diffusivity [29]. Particular interest has been shown in predicting technical properties [30], most of which were devoted to concrete compressive strength prediction.

The complexities of predicting concrete strength through machine learning were first articulated by Yeh et al. [31] in 1998. They experimented with seven input variables using artificial neural networks (ANN) and linear regression to forecast the strength of high-strength concrete. While their model was trained on a vast array of concrete samples, these were not scrutinised for content. Their study considered concrete samples in the maturing phase, including those as young as three days old, which may have led to skewed results.

In 2003, this subject was further refined by Seung-Chang Lee [32], who employed a unique, modular network architecture consisting of five ANNs. Each ANN represented concrete at various stages of maturation up to its maximum strength. Lee used the parameter condensation technique to ascertain the number of neurons in the input layer. Despite claiming his condensation and weighing techniques as beneficial for optimal network performance, the practicality of his ANN model, which illustrates the maturation process from pouring to full strength, is questionable. From an engineering perspective, attention should be devoted to concrete that has achieved full or near-full strength.

In 2005, Hola, J. and Schabowicz, K. [33,34] introduced a novel nondestructive approach for assessing concrete strength. Rather than relying on the concrete mix composition, they trained their ANN model on data obtained from nondestructive concrete testing tools. Their database incorporated ultrasonic wave velocity, reflection number, hardness, pull-out strength, concrete age, and bulk density. To evaluate their lab results, they experimented on concrete compressive strength samples with a 28-day strength ranging from 24 to 105 MPa. Using the Levenberg–Marquardt training method, they developed the ANN with eight hidden neurons within one layer. The authors posited that the average compressive strength comparison between the ANN and nondestructive tests was comparable.

In 2006, a neural-expert system for predicting the compressive strength of high-performance concrete was proposed by Gupta et al. [35]. They focused on training the algorithm through example inferences, employing a multilayer ANN trained with generalised backpropagation for interval training patterns. They also used input variables from unrelated metrics, such as curing time, that were unrelated to the recipe. However, these strategies may lead to algorithm training based on insignificant patterns and unclear results. The use of a neural-expert system for concrete compressive strength prediction was also explored by Dac-Khuong Bui et al. [36] with a focus on the practical application of this method.

The advent of deep learning in this field was introduced by Fangming Deng et al. [37] in 2018. They prepared a database of recycled concrete samples for algorithm training. They chose not to train the algorithm on the concrete mix composition with direct amounts of individual components, but they called deep features on several ratios. This approach was emulated in the current study with the inclusion of feature scaling. Deng and his team used Softmax regression to identify a suitable prediction model. They claimed that deep learning compared to ANN, provided better generalisation capabilities, superior efficiency, and greater precision. However, these claims were not definitive and warranted further research. Given that convolution neural networks are computationally costly, the authors used a limited database of 74 samples compared to 741 in the current study. This limited sample size might lead to underfitting, implying a model that does not fully capture the modelled phenomenon. A comparable level of accuracy between artificial neural networks and deep neural networks was reported by Hosein Naderpour et al. [38] in 2018.

Ziolkowski, P. et al. [39] introduced an algorithm in 2019 that assists in designing a concrete mix by predicting the strength based on the mix composition. While this algorithm accurately predicted the strength of the concrete mix, it underperformed in the high-strength spectrum of 40 MPa and beyond and was insufficient in predicting the properties of mixtures with additives and admixtures. Furthermore, it neglected other essential parameters that contribute to concrete’s performance, such as durability, which is essential for maintaining structural service quality over time [40].

In a publication from 2020, Nunez, I. and his team [41] shared insights on using machine learning to accurately forecast the compressive strength of recycled aggregate concrete, consequently refining the concrete mix design process. The researchers recognised the critical importance of an effective optimisation method for concrete mix design in light of the inherent variability and the absence of reliable compressive strength prediction formulae for recycled aggregate concrete. Three innovative machine learning models were developed in their study, specifically the Gaussian process model, the recurrent neural network model, and the gradient-boosted regression tree model. Based on their findings, they reported superior predictive outcomes, particularly with the gradient-boosted regression trees model.

Another noteworthy contribution from the same year is a study by Marani, A. and his colleagues [42], who explored the use of machine learning to forecast the compressive strength of ultra-high-performance concrete. Their algorithm was trained on a comprehensive dataset of 810 samples from freely accessible resources, encompassing 15 variables as input data. Rather uniquely, they capitalised on their dataset to generate 6513 records, a substantial number of synthetic data samples, using tabular generative adversarial networks. The wealth of data facilitated a more robust training of their machine learning model. Upon evaluation, the model trained with synthetic data yielded exceptional predictive performance when assessed with the primary dataset.

In 2021 Ziolkowski, P. et al. [43] introduced a new adaptive machine learning method that more precisely estimates the compressive strength of concrete based on the composition of its primary ingredients. Unlike previous models that had mixed success in forecasting concrete strength and struggled to encapsulate the variability inherent in current concrete mixes, this method incorporated two observations for each concrete batch in the model. The authors built this machine learning model using a deep neural network architecture and trained it on a comprehensive database of concrete recipes before translating it into a mathematical formula. The algorithm was tested on four concrete mix recipes calculated using contemporary design methods such as Bolomey and Fuller, with the findings revealing that this new algorithm outperformed nonadaptive models trained on the same dataset.

Adil, M. et al. [44] investigated the effect of the number of neurons and layers in ANN for generalised concrete mix design. They developed an ANN with 17 inputs and five outputs related to the concrete mix’s composition and properties. The authors proposed optimising the network with one or two hidden layers. It represented a significant departure from previous work, where concrete’s technical parameters were predicted based on the composition ratio.

Feng, W., in their paper [45] explores the mechanical characteristics of rubber-modified recycled aggregate concrete (RRAC). The authors utilised machine learning (ML) models to predict these thermomechanical properties of RRAC, employing a unique algorithm called the beetle antennae search (BAS) to tune the hyperparameters of these models. Four ML models were tested: random forest, logistic regression, multiple linear regression, and backpropagation neural network (BPNN). Among them, BPNN yielded the most accurate and reliable UCS and peak strain predictions, suggesting that ML models, particularly BPNN, can serve as robust tools for predicting the properties of sustainable construction materials such as RRAC. This study highlights the potential of RRAC in sustainable construction and the effective use of ML models in predicting its performance.

Tavares, C. et al., in their two-part study [46,47], proposed an innovative method that utilises machine learning (ML) for the optimised mixture design of ultra-high-performance concrete (UHPC). This methodology presents an attractive alternative to resource-consuming experimental runs by employing orthogonal arrays for data collection, which could enable ML design optimisation. The researchers used an ensemble of ML techniques, specifically random forest and k-nearest neighbours, to create performance density diagrams (PDDs). These diagrams serve as an intuitive tool to demonstrate the trade-offs between mix proportions and mechanical performance of UHPC, providing practical assistance to designers in the construction industry. Their research has shown promising results, where the PDDs effectively predicted the behaviour of most mixtures in the test set. This method facilitated the design of a UHPC mixture averaging 155 MPa at age 56 days, maintaining the fine-aggregate-to-cementitious-material ratio above the unit. It represents a substantial advancement in developing mix design tools for UHPC, leading to cost and eco-efficiency improvements. Notably, this methodology was further extended in the second part of their study to allow simultaneous evaluation of performance, cost, and carbon footprint. This approach lays a foundation for the broader adoption of ML techniques in sustainable construction and the development of mix designs for UHPC.

Endzhievskaya, I.G. et al. [48] presented a study on road concrete’s physical and mechanical characteristics. The authors employed machine learning techniques, specifically a random forest and decision trees. These methods were advantageous due to their ease of use, minimum hyperparameters for tuning, and the ability to predict with low errors. Their findings indicate that components such as air-entraining additives and specific sizes of crushed stone contribute significantly to improving compressive and bending strengths. Machine learning’s predictive accuracy demonstrated its potential in optimising road concrete mixtures, enhancing road surfaces’ quality and service life.

The study presented by Taffese, W.Z. and Espinosa-Leal, L. [49] stands out in concrete properties prediction. Their research leverages machine learning, specifically decision tree-based ensemble methods, to develop multitarget models predicting compressive strength and nonsteady-state chloride migration coefficients (D_nssm_) of concrete. This work’s novelty lies in developing a single model that simultaneously predicts two crucial concrete properties, compressive strength and D_nssm_. The gradient boosting model demonstrated the most impressive prediction accuracy, yielding the mean absolute error (MAE) of 6.683 and 1.363, mean squared error (MSE) of 83.369 and 3.712, and root mean squared error (RMSE) of 9.131 and 1.927 for compressive strength and D_nssm_, respectively. The authors stress the necessity to expand the model with comprehensive datasets encompassing a wider range of concrete properties to improve its versatility.

## 3. Materials and Methods

### 3.1. Essentials

Machine learning models can be used to predict concrete’s technical properties based on the mix composition. This study tries to determine the impact of increasing the computational complexity of the artificial neural network on the model’s performance. The quantity of layers is one of the deep neural network (DNN)’s [50,51] features that represents a critical determinant of model complexity and its inherent capability to discern and replicate complex patterns embedded in the data. This aspect forms the basis of the term “deep” within deep learning, where an increased number of layers, representing greater depth, facilitates the modelling of progressively intricate and abstract features. Each layer within a DNN can be conceptualised as performing successive transformations of the raw input into higher-level, abstract features. For instance, in applying convolutional neural networks (CNNs) [52,53,54], commonly used for image recognition tasks, initial layers may decipher basic, low-level features such as edges and colours. As the depth of the network increases, subsequent layers amalgamate these rudimentary features to detect more abstract patterns, encompassing shapes and, ultimately, entire objects or scenes.

However, while an increased depth can enable a model to learn more complex representations, it poses new challenges. The augmentation in the number of layers directly expands the model’s parameters, thereby escalating the risk of overfitting. Overfitting manifests when a model excessively adapts to the training data, compromising its ability to generalise to unseen data effectively [55,56,57]. This becomes particularly problematic when the volume of available training data is limited compared to the complexity of the model. Furthermore, training highly deep networks introduces additional technical difficulties. One notable issue is vanishing or exploding gradients, which can decelerate training or result in suboptimal solutions [58,59]. Techniques such as parameter initialisation, batch normalisation, and incorporating residual connections have been suggested to alleviate these concerns. Consequently, the selection of an optimal layer quantity necessitates a delicate balancing act, taking into account the trade-off between a network’s capacity to model intricate patterns (which tends to increase with depth) and the potential challenges associated with overfitting and training difficulties. Strategies such as early stopping, dropout, and using validation sets can help to manage the risks inherent to increased depth [60,61,62]. Despite these challenges, the capacity to train deep networks remains a pivotal factor propelling recent advancements in artificial intelligence and machine learning.

**Figure 1:**
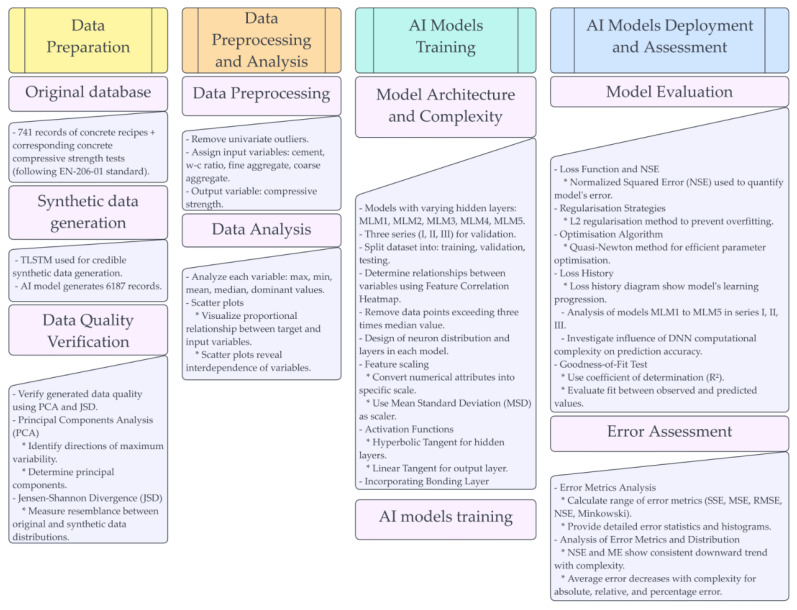
Flowchart showing the procedures described in this study.

The analysis adopted a classical approach involving the construction of a model that will estimate the concrete compressive strength determined by the quantitative concrete mix composition. The prepared analysis used a database from previous studies [39,43], which contains several hundred records of concrete recipes, along with corresponding destructive compressive strength tests on normalised samples in the laboratory. The number of records has been increased by using a dedicated AI model to generate reliable synthetic data [63,64,65]. The database used contains recipes for the concrete mix composition, which were designed as intended for incorporation into various concrete elements. At the same time, differentiation of dimensions, functions, and purpose is assumed here. Some recipes contained admixtures for various purposes, such as workability improvers, plasticisers or setting retarders. It is taken for granted that the concrete production process employed met the necessary quality standards. However, due to varying design requirements and the use of different admixtures, some differences between formulations may be difficult to quantify. Therefore, the procedure of removing univariate outliers as multiples of the standard deviation was used, described in detail later in the paper. Individual components of concrete mixes and the water–cement ratio were assigned input variables, while the concrete compressive strength was treated as the output variable. The presented study, along with many studies in the literature, focuses on predicting one of the main technical properties of concrete, which is concrete compressive strength. However, it should be noted that many other technical properties affect the final behaviour of concrete, especially at various stages of the technological production process. The quality of this process is also essential, which is influenced by factors such as the curing process [66,67] or the concrete pouring temperature [68]. Figure 1 shows a flowchart illustrating the research procedures outlined in this investigation.

### 3.2. Data Processing

The database used in this research contains 6187 records, generated using a dedicated AI model from the original database that contained 741 records of concrete recipes [39,43], along with corresponding compressive strength tests conducted under laboratory conditions on standard samples according to PN-EN: 206 [23]. This set has six variables, as follows: f_ck_—concrete compressive strength (MPa), C—cement (kg/m^3^), W—water–cement ratio (-), FA—fine aggregate (kg/m^3^), CA—coarse aggregate (kg/m^3^). These synthetic data do not create new knowledge but helps to achieve better robustness of the AI model. The parameters utilized have been showcased in Table 1. A fundamental statistical analysis was prepared for each variable in the analysed dataset. Table 2 shows each variable’s maximal, minimal, mean, median, and dominant values.

**Figure 2:**
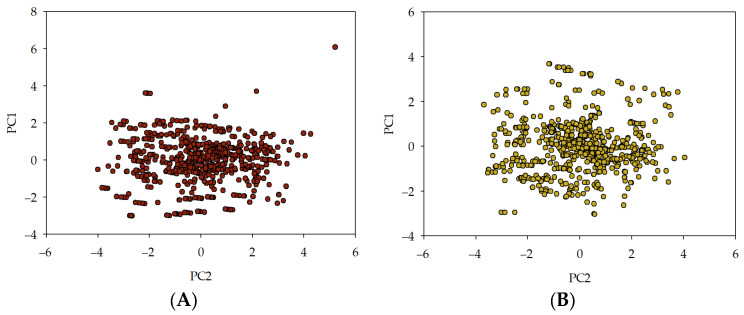
Principal component analysis: training data (**A**), synthetic data (**B**). Training data are the data used to generate synthetic data.

The tabular long short-term memory (TLSTM) model [69,70] was used to generate credible synthetic data. The experiments were carried out to validate the excellence of the produced data, specifically a principal components analysis (PCA) [71,72] and Jensen–Shannon divergence (JSD) [73,74,75,76]. In order to verify the statistical integrity of deeper, multifield distributions and correlations, a comparative analysis was performed using PCA computed first on the original data, then on the synthetic data. The basis of PCA lies in capturing the essential shape of all the features in a few key features, referred to as the principal components. Consider a dataset with just two columns, as graphed below for illustrative purposes. PCA can be visualised as an exercise in fitting an ellipsoid to the data, where the axis of the ellipsoid, signifying the directions of maximum variability in the data, represents the principal components. In a more complex multidimensional scenario, the objective of PCA becomes analogous to rotating an object in hand to achieve a view of maximal width, which is then determined to be the first principal component. Subsequent rotations, while maintaining horizontal steadiness, aim to achieve the view of maximal height, thus determining the second principal component. The approach is structured around identifying the axis with maximum variability, always maintaining perpendicularity to the previously chosen axis. Consequently, the newly created dimensions encapsulate the essence of the fundamental shape of the data. The quality of synthetic data can be assessed by evaluating the distributional distance between the principal components in the original data and those in the synthetic data. The proximity of the principal components directly influences the quality of synthetic data, with closer principal components yielding better quality. Given the ubiquity of PCA in machine learning for dimensionality reduction and visualisation, this score provides an immediate assessment of the utility of the obtained synthetic data for machine learning applications. The approach hence aims to measure the statistical integrity of the synthetic data by evaluating its conformance to the structure encapsulated in the principal components of the original data. The results of PCA are presented in Figure 2.

**Figure 3:**
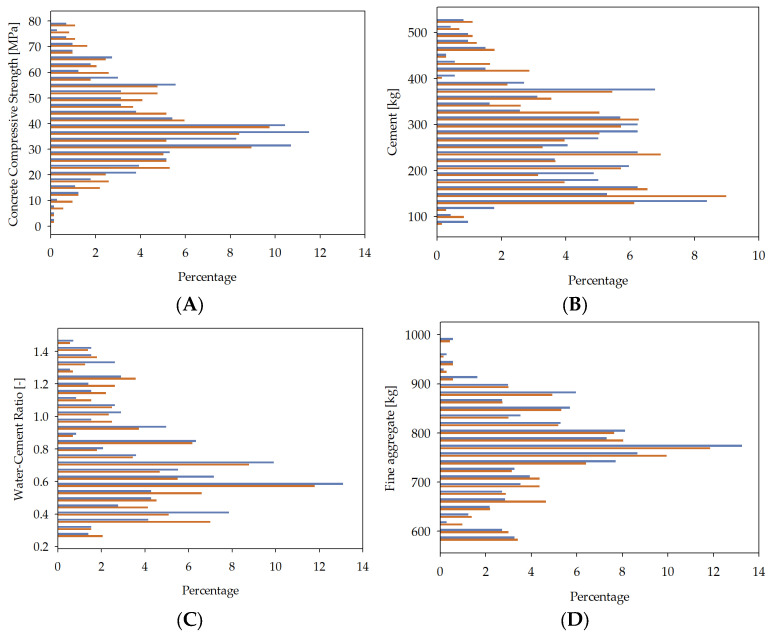

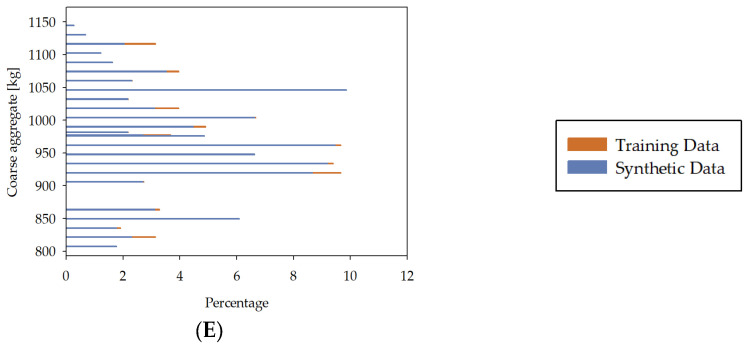
Field distribution comparisons: concrete compressive strength (**A**), cement (**B**), water–cement ratio (**C**), fine aggregate (**D**), coarse aggregate (**E**). Purple bars correspond to training data. Green bars correspond to synthetic data. Vertical axes are percentages.

JSD represents the degree of resemblance between the field distributions of the original and synthetic data. It is a method commonly applied for comparing two distributions. The average Jensen–Shannon divergence value across all fields inversely correlates with the data quality, with lower value indicating higher quality. A visual comparison of the original and synthetic field distributions is facilitated by presenting bar charts in Figure 3.

The compressive strength of concrete was assessed in a laboratory setting utilising standardised samples under the EN-206-01 standard [77]. The samples in question, standardised as per the specifications, were cylinders with a diameter of 15 cm and a hight of 30 cm and cubes with a side length of 15 cm. The results were presented as the compressive strength of cylindrical samples, while the results from the cubic samples were converted according to the standard mentioned above [77] to represent the strengths obtainable from cylindrical samples. The samples were fabricated using ordinary portland cement, and the sand employed was free from clay contamination. As per the EN-206-01 standard [77], the strength was examined after 28 days, generally when the concrete attained full strength. It should be emphasised that the time required for concrete to achieve full strength mainly depends on the type of cement used, and for some types of cement, this period may be longer or shorter. However, it was assumed that the cement used in the mixture did not result in any reduction or extension of the time to achieve strength. To standardise the investigation, samples without full strength were excluded from the dataset. The quantity of records mentioned above is devoid of such samples. Due to the fact that all ANN has been trained on specific datasets, it is advisable to operate within the maximum values of the model’s input parameters and preferably avoid deviant areas. In some cases, input parameters that fall outside the maximum values of the model can lead to unreliable or inaccurate results.

**Figure 4:**
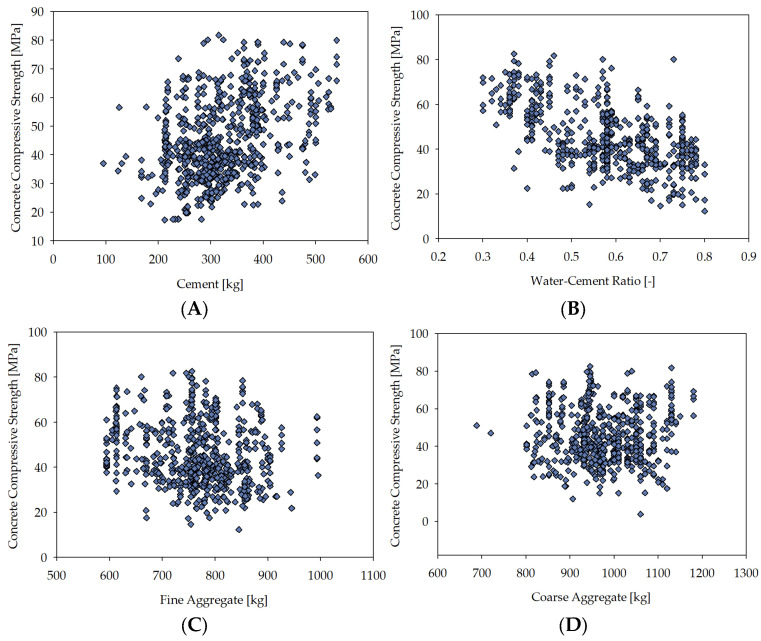
The scatter plots depict the relationship between the input variables and the target variable. The vertical axis represents the compressive strength of concrete in MPa, while the horizontal axis represents input variables: (**A**) cement (kg/m^3^), (**B**) water–cement ratio (-), (**C**) fine aggregate (kg/m^3^), (**D**) coarse aggregate (kg/m^3^).

Figure 4 displays scatter plots that elucidate the proportional relationship between targeted and input variables. The utility of scatter plots as a comprehensive method for scrutinising the interdependence of variables is well-established [78]. It is a visually striking demonstration of the nexus between these two variable categories. Owing to the many possible combinations, only a select number of examples involving target variables were furnished for illustrative purposes. The said plots specifically cater to the objective variable corresponding to the concrete compressive strength.

### 3.3. Training, Testing, and Model Selection

**Figure 5:**
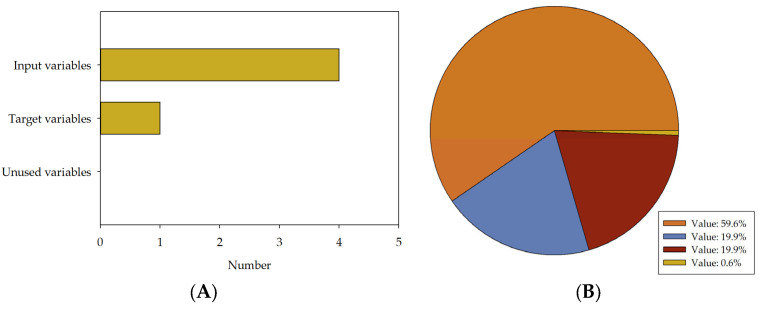
Number of input, target, and unused variables in final models and division of subsets. The diagram includes a variable bar chart (**A**) and a sample pie chart (**B**).

The above database was used to train a series of deep artificial neural network models. The goal of the analysis is to investigate the influence of the computational complexity of DNNs on the accuracy of predicting technical parameters of concrete. Created models, given the quantitative composition, can estimate the compressive strength of concrete. In the analysis below, five neural network models of varying computational complexity, differentiated by the number of hidden layers (MLM1, MLM2, MLM3, MLM4, MLM5), were compared, repeating the entire process in three series (I, II, III) for validation purposes. From the least complex model, MLM1 has two hidden layers, to MLM5, which has six hidden layers. The number of neurons in a typical hidden layer is four. Each model has five parameters, four input variables, and one output variable. For effective training of deep neural networks, the dataset is typically divided into three independent parts: training, validation (or selection), and testing. This is a standard procedure applied in deep learning [79,80]. The training set is used to optimise the parameters of the neural network. The validation set allows its effectiveness to be assessed during the learning process and to choose the best model, while the test set is used to evaluate the final performance of the model. An outlier elimination procedure was implemented in the datasets under investigation where any data point exceeding three times the median value of each variable, calculated from the dataset’s centre, was excluded. These exclusion criteria, formulated to target univariate outliers, were employed to safeguard the precision and dependability of the subsequent statistical analyses [81,82]. Due to the influential effect of univariate outliers, the potential distortion of results could yield a misinterpretation of the dataset’s actual characteristics. Thus, outlier removal enhances the sample’s representativeness, contributing to more robust and reliable results. It is essential to acknowledge that this procedure, despite potentially impacting the sample size, is essential in affirming this study’s validity. The applied process ensures the statistical integrity of the analyses, reinforcing the reliability of this study’s conclusions. The used dataset was allocated as follows: 59.6% of the records (3689) were assigned to the training set, 19.9% (1229 records) to the selection set, 19.9% (1231 records) to the test set, and 0.6% (38 records) were unused. The number of input, target, and unused variables in the final models and the division of subsets are presented in Figure 5.

**Figure 6:**
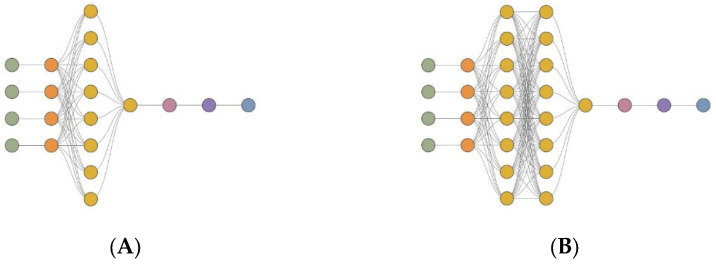

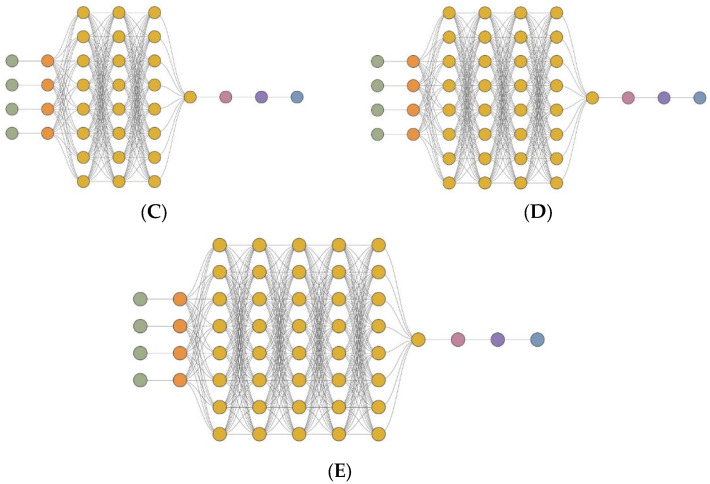
The topology of the deep neural network (DNN) for each model is presented as follows: MLM1 (**A**)**,** MLM2 (**B**), MLM3 (**C**), MLM4 (**D**), and MLM5 (**E**). The figure shows the DNN architecture, which includes input neurons (green), scaling neurons (orange), hidden neurons (yellow), descaling neurons (red), bonding neurons (purple), and output neurons (blue).

The architecture of the first model, MLM1, consists of 20 neurons, including four input neurons, nine neurons spread over two hidden layers, with four neurons in one scaling layer, one neuron in the descaling layer, one neuron in the bonding layer, and one neuron in the output layer. The architecture of the second model, MLM2, consists of 28 neurons, including four input neurons, 17 neurons spread over three hidden layers, with four neurons in one scaling layer, one neuron in the descaling layer, one neuron in the bonding layer, and one neuron in the output layer. The architecture of the third model, MLM3, consists of 36 neurons, including four input neurons, 25 neurons spread over four hidden layers, with four neurons in one scaling layer, one neuron in the descaling layer, one neuron in the bonding layer, and one neuron in the output layer. The architecture of the fourth model, MLM4, consists of 44 neurons, including four input neurons, 33 neurons spread over five hidden layers, with four neurons in one scaling layer, one neuron in the descaling layer, one neuron in the bonding layer, and one neuron in the output layer. The architecture of the fifth model, MLM5, consists of 52 neurons, including four input neurons, 41 neurons spread over six hidden layers, with four neurons in one scaling layer, one neuron in the descaling layer, one neuron in the bonding layer, and one neuron in the output layer. Figure 6 shows the architectures of the models analysed in this research.

The data features, represented as input variables, are assigned to the input neurons of the neural network structure, while the output neuron is connected to the target variables. To enhance the model’s effectiveness, a method known as feature scaling was implemented across all models. This process entails converting the numerical attributes of the data into a specific scale [83]. This scaling and the subsequent descaling were carried out using the mean standard deviation (MSD) as a scaler [84]. The models maintain consistent activation functions, with the hyperbolic tangent [85,86,87] being used for the hidden layers and the linear tangent [87] for the output layer. A bonding layer was also incorporated into the models. The constructed models were meticulously calibrated to minimise the associated loss function. The quantification of the model’s error in computing the index loss was executed utilising the normalized squared error (NSE). It is to be noted that lower NSE values are indicative of a model’s superior predictive capabilities. Conversely, values tending towards one highlight the model’s weaker predictive potential, while values close to zero signify a commendable predictive performance. To further enhance the performance of our model and inhibit the potential for overfitting or underfitting, the adoption of regularisation strategies was necessitated. Specifically, the L2 method [88,89,90] was instituted as our chosen regularisation function. This regularisation phase is instrumental in tuning the model by minimising the adjusted loss function. It contributes towards mitigating biases and, consequently, facilitates more precise predictions. It is pertinent to underscore the importance of the regularisation step in the model-building process, as it aids in ensuring that the model is absorbing the intrinsic patterns and relationships within the data instead of merely reproducing the training data. In successfully preventing overfitting and underfitting, a model is deemed accurate and generalisable, rendering it suitable for application to novel, unseen data. In this context, the efficacy of the L2 regularisation method [88,89,90] is particularly pronounced. It appends a penalty term to the loss function proportional to the square of the magnitude of the network parameters’ weights. As a result, the weights are driven towards zero, facilitating the generation of smaller, simpler models less susceptible to overfitting.

**Figure 7:**
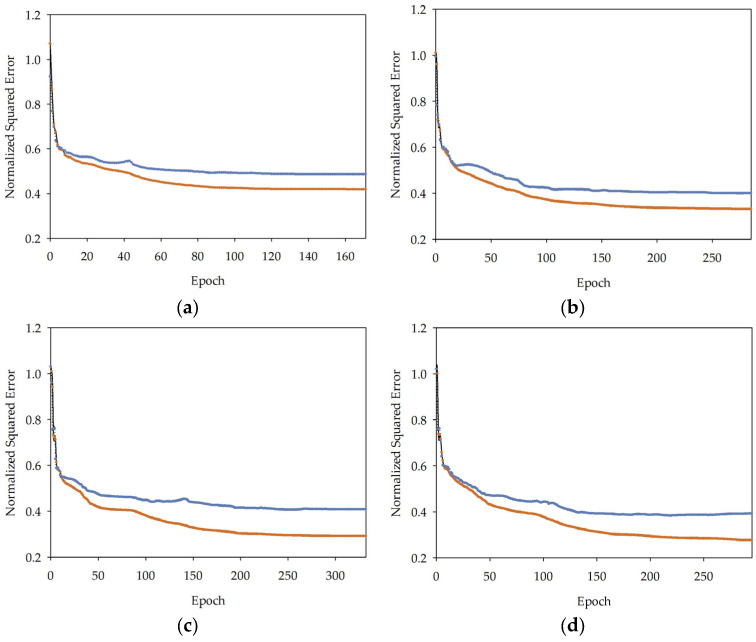

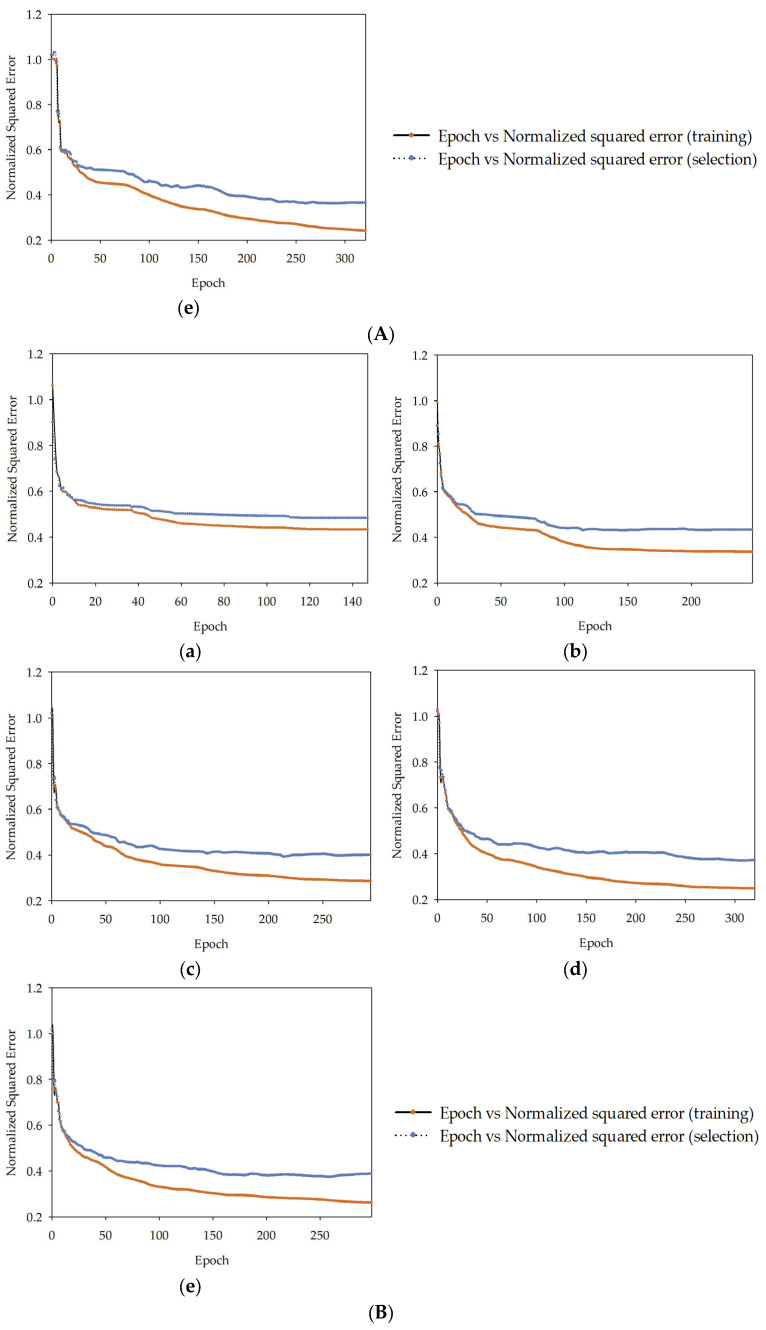

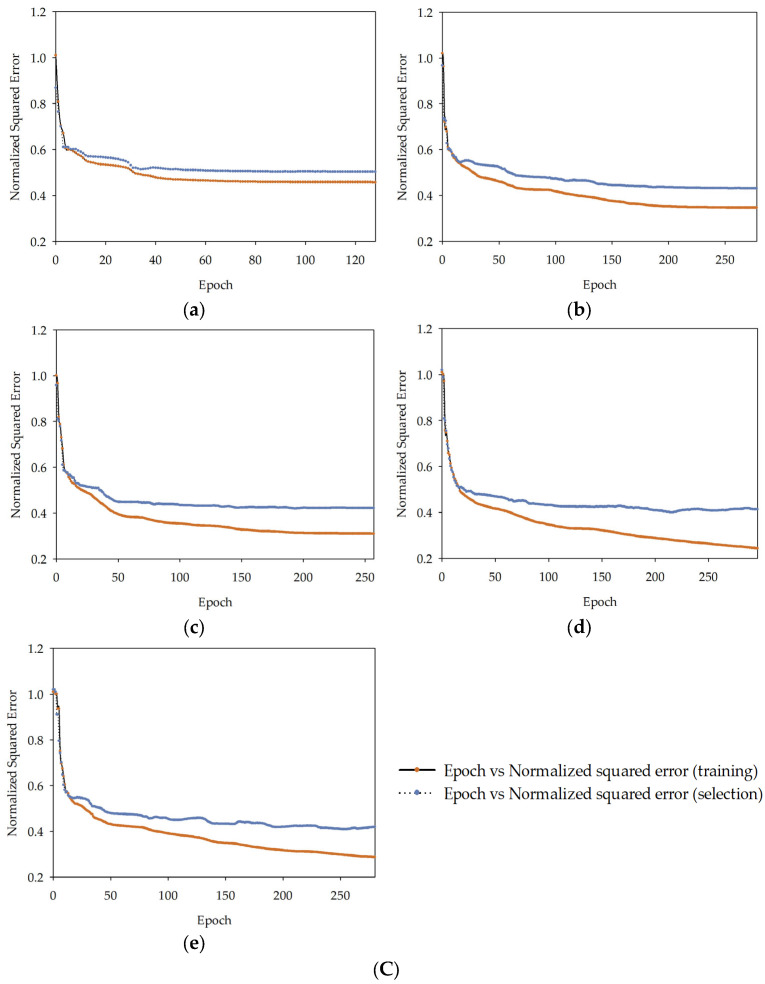
(**A**) Loss history diagram for specific models in series I: MLM1 (epoch from 1 to 171) (**a**), MLM2 (epoch from 1 to 285) (**b**), MLM3 (epoch from 1 to 332) (**c**), MLM4 (epoch from 1 to 295) (**d**), MLM5 (epoch from 1 to 321) (**e**). (**B**) Loss history diagram for specific models in series II: MLM1 (epoch from 1 to 147) (**a**), MLM2 (epoch from 1 to 248) (**b**), MLM3 (epoch from 1 to 294) (**c**), MLM4 (epoch from 1 to 320) (**d**), MLM5 (epoch from 1 to 298) (**e**). (**C**) Loss history diagram for specific models in series III: MLM1 (epoch from 1 to 128) (**a**), MLM2 (epoch from 1 to 278) (**b**), MLM3 (epoch from 1 to 257) (**c**), MLM4 (epoch from 1 to 296) (**d**), MLM5 (epoch from 1 to 280) (**e**).

The quasi-Newton method [91,92] was employed as the optimisation algorithm in the present study. The quasi-Newton method is a popular choice for optimisation algorithms due to its efficiency and effectiveness in tackling large-scale optimisation problems [92]. It does so by using first-order derivative information to build up an approximation of the Hessian matrix [93], which represents the second-order partial derivatives of the objective function [91]. This method has shown substantial success in solving nonlinear optimisation problems that arise in diverse applications, thanks to its robustness and ability to converge to the solution more quickly than traditional gradient descent algorithms. The quasi-Newton method’s effectiveness is enhanced by its ability to handle functions that are not necessarily smooth, making it a versatile tool in optimisation [94]. Employing this algorithm in the current study was an integral part of the process, allowing for the efficient optimisation of the model’s parameters. The resulting loss history for the model and series are presented in Figure 7A–C (for series I, II, and III, respectively), illustrating the model’s learning progression throughout the training process. The loss history represents how the loss function of a machine learning model changes over the course of its training and selection process. The loss function quantifies the discrepancy between the predicted outputs of the model and the actual target values, and the goal of training is to minimize this loss. The adopted training strategy proved to be highly effective in optimising the model’s performance, providing the desired level of accuracy while minimising computational resources.

It should be noted that the models were trained on a specific set of data. Therefore, when using the models, one should operate within the values indicated in Table 2 as minimal and maximal. The following research does not consider the impact of using additives and admixtures on concrete. The usable range for the water–cement ratio extends from approximately 0.3 to beyond 0.8. A proportion of 0.3 results in highly rigid consistency (unless superplasticisers are employed), while a ratio of 0.8 yields concrete that is damp and lacking in strength [95,96]. All records outside the range water–cement 0.3–0.8 have been removed from the dataset.

### 3.4. Results and Discussion

**Figure 8:**
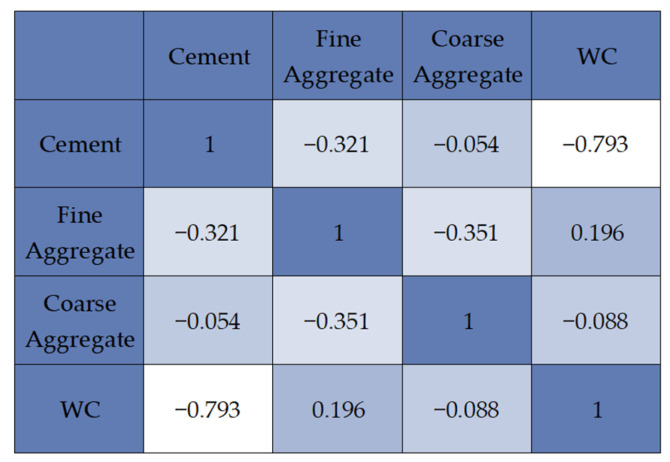
Feature correlation heatmap.

In the following study, five models of deep artificial neural networks with varying degrees of computational complexity were analysed, named consecutively MLM1, MLM2, MLM3, MLM4, and MLM5, starting from the least complex network to the most complex, in three series I, II, III. First, a feature correlation analysis was prepared to determine precise relationships between individual variables. The result of the analysis is a feature correlation heatmap visible in Figure 8.

The feature correlation heatmap reveals the relationships of individual variables, where a value closer to 1.0 indicates a stronger correlation [97]. One can notice that the input variables related to the water–cement ratio and the cement content have the strongest association with the output variable. The relationships of other input variables with the output variable are much weaker, with the amount of fine aggregate having a more significant impact than water content and water content having a greater impact than the amount of coarse aggregate. It can be observed that the impact of the water–cement ratio and the quantity of cement on the strength of concrete is evident. Several research papers in the literature have affirmed the crucial role of these factors in determining the compressive strength of concrete [98].

**Figure 9:**
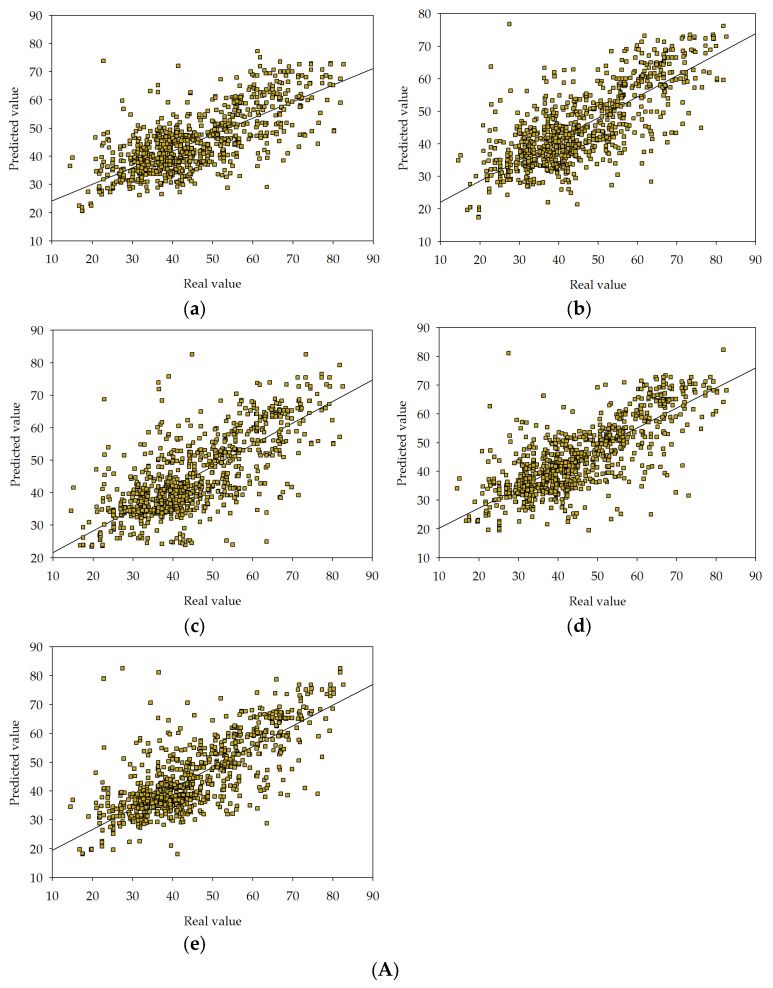

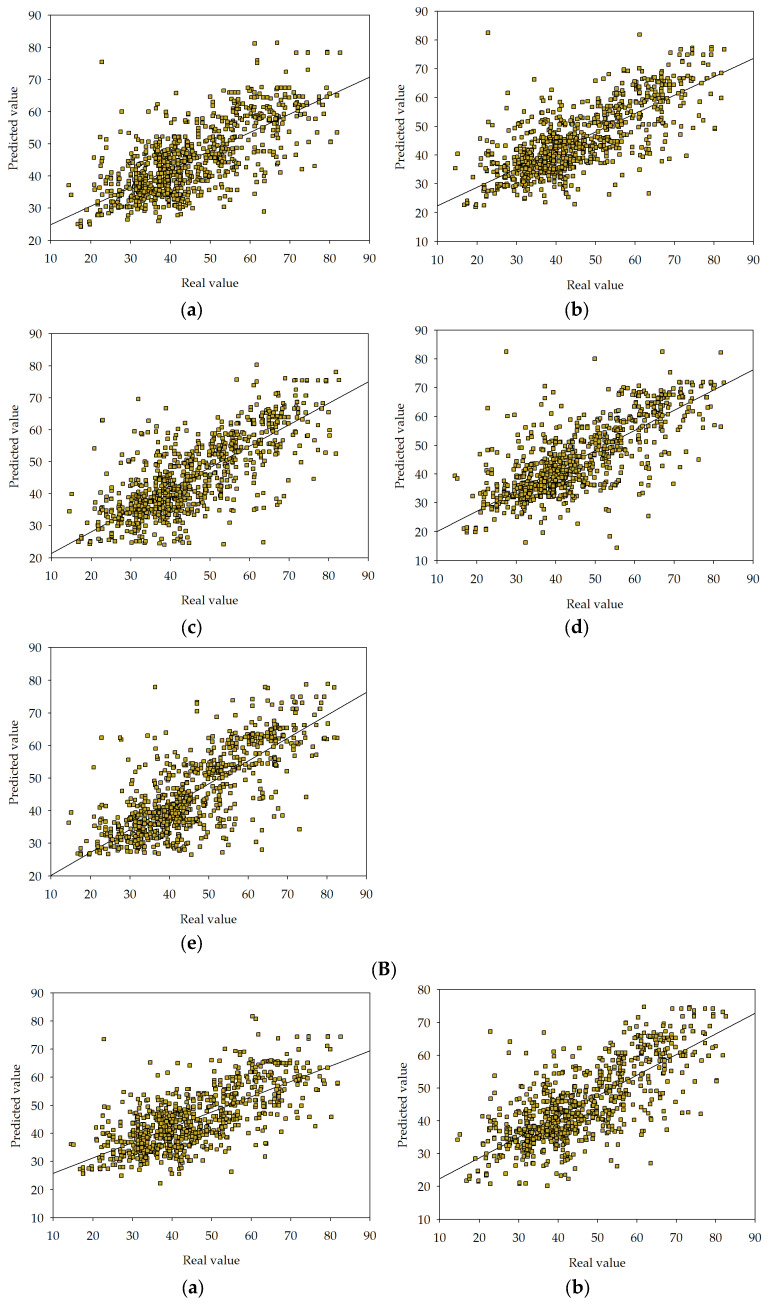

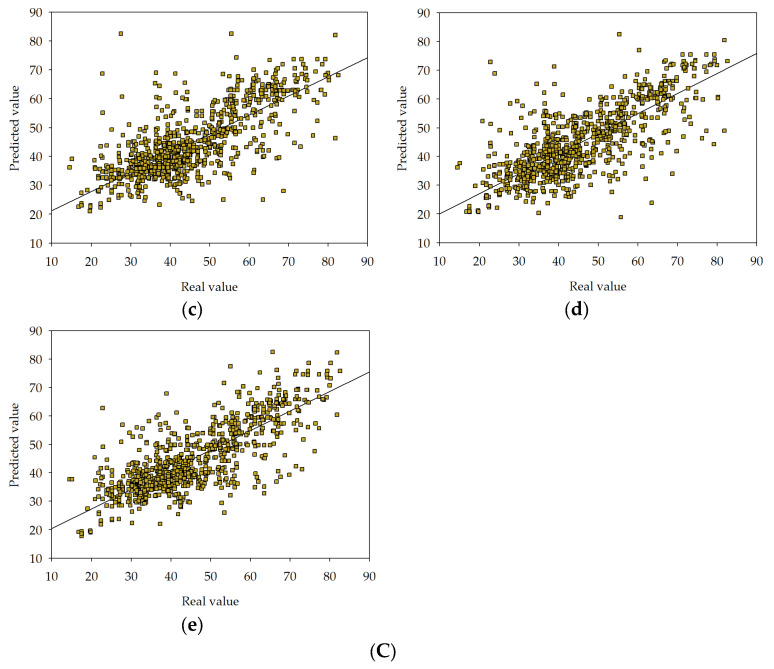
(**A**) Goodness-of-fit chart for MLM1 (**a**), MLM2 (**b**), MLM3 (**c**), MLM4 (**d**), MLM5 (**e**) in series I. The chart shows predicted value of target variable versus real one. (**B**) Goodness-of-fit chart for MLM1 (**a**), MLM2 (**b**), MLM3 (**c**), MLM4 (**d**), MLM5 (**e**) in series II. The chart shows predicted value of target variable versus real one. (**C**) Goodness-of-fit chart for MLM1 (**a**), MLM2 (**b**), MLM3 (**c**), MLM4 (**d**), MLM5 (**e**) in series III. The chart shows predicted value of target variable versus real one.

Each of the models was subjected to a goodness-of-fit test [99,100,101,102,103]. This assessment provides a means to quantify the discrepancy between observed values and those predicted by the model. A standard metric used to gauge the goodness-of-fit in scientific investigations is the coefficient of determination, denoted as R^2^ [104,105]. This parameter is utilized to measure the extent of disparity between observed values and projected predictions. More specifically, R^2^ determines the fraction of this discrepancy that can be accounted for by the model. In a scenario where the model fit is ideal, resulting in output values perfectly matching the target values, the R^2^ coefficient would equate to one. Figure 9A–C provide a detailed visual representation of a goodness-of-fit analysis for series I, II, and III, utilising the statistical measure known as the coefficient of determination (R^2^).

**Figure 10:**
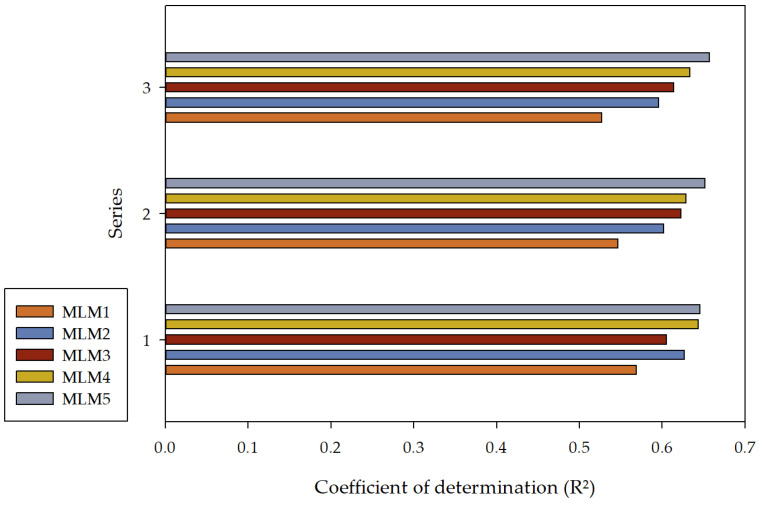
The coefficient of determination (R^2^) values for MLM1, MLM2, MLM3, MLM4, and MLM5 in series I, II, and III.

In the considered issue, a series of R^2^ values were calculated for the target variable f_ck_ in the individual models and series. In series I, it was 0.5691 for MLM1, 0.6268 for MLM2, 0.6053 for MLM3, 0.6438 for MLM4, and 0.6453 for MLM5. In series II, it was 0.5467 for MLM1, 0.6017 for MLM2, 0.6227 for MLM3, 0.6285 for MLM4, and 0.6514 for MLM5. In series III, it was 0.5272 for MLM1, 0.5959 for MLM2, 0.6136 for MLM3, 0.6337 for MLM4, and 0.6571 for MLM5. The values of the coefficient of determination (R^2^) for each model are shown in Figure 10.

**Figure 11:**
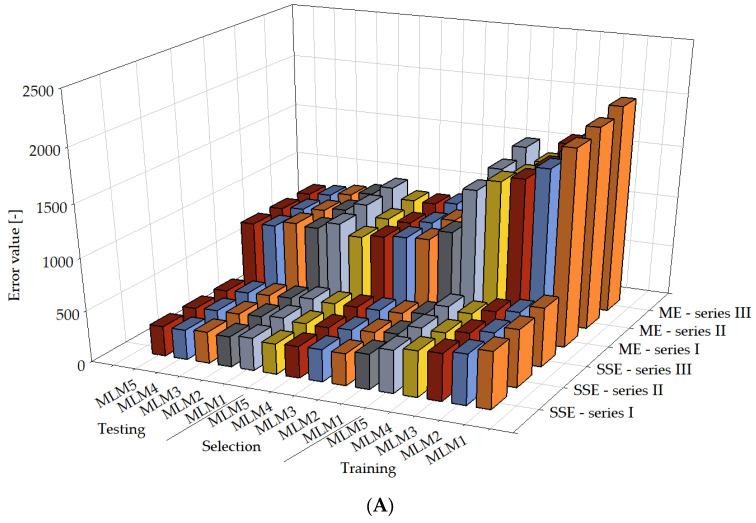

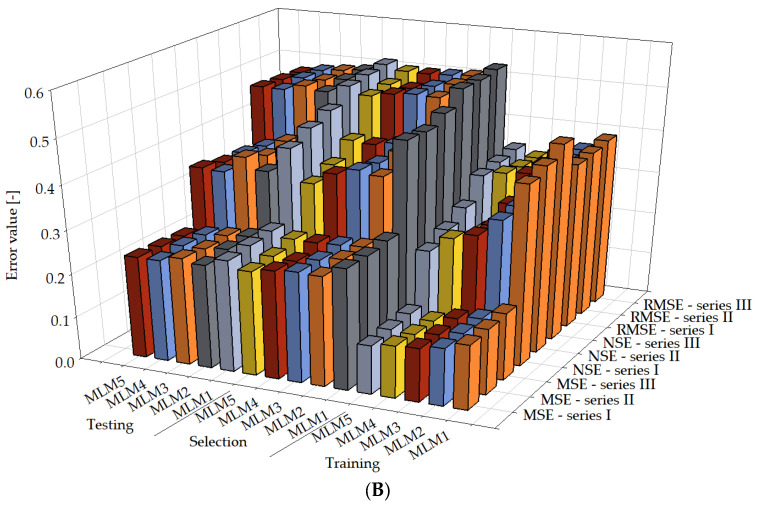
Error metrics for MLM1, MLM2, MLM3, MLM4, MLM5 in series I, II, and III. Error metrics used: mean squared error (MSE) (**B**), Minkowski error (ME) (**A**), normalized squared error (NSE) (**B**), root mean squared error (RMSE) (**B**), sum squared error (SSE) (**A**).

It can be observed that a reasonably good performance of the created models was achieved. Simultaneously, with the increased computational complexity, a higher R^2^ value can be noticed in more complex models, suggesting that these models exhibit better predictive capabilities than less complex models. This observation is evident in three series, except for series I, where the MLM2 model has a higher R^2^ coefficient value than the MLM3 model. This deviation was not observed in the remaining series and may result from various reasons. An exhaustive analysis of the model’s errors was performed by computing a range of error metrics across each series. This analysis included metrics such as mean squared error, Minkowski error, normalized squared error, root mean squared error, sum squared error [106,107,108,109]. Furthermore, a detailed report outlining the minimum and maximum values and the mean and standard deviation for absolute, relative, and percentage errors of the model concerning the test data was provided. Histograms were constructed for the test subset to obtain a more tangible understanding of the distribution of errors in the model. The outcomes of this analysis provide a rigorous evaluation of the model’s accuracy and precision, illuminating potential avenues for further refinement. Table 3 and Figure 11 present individual error metrics for each model, divided into respective subsets, for series I, II, and III.

**Figure 12:**
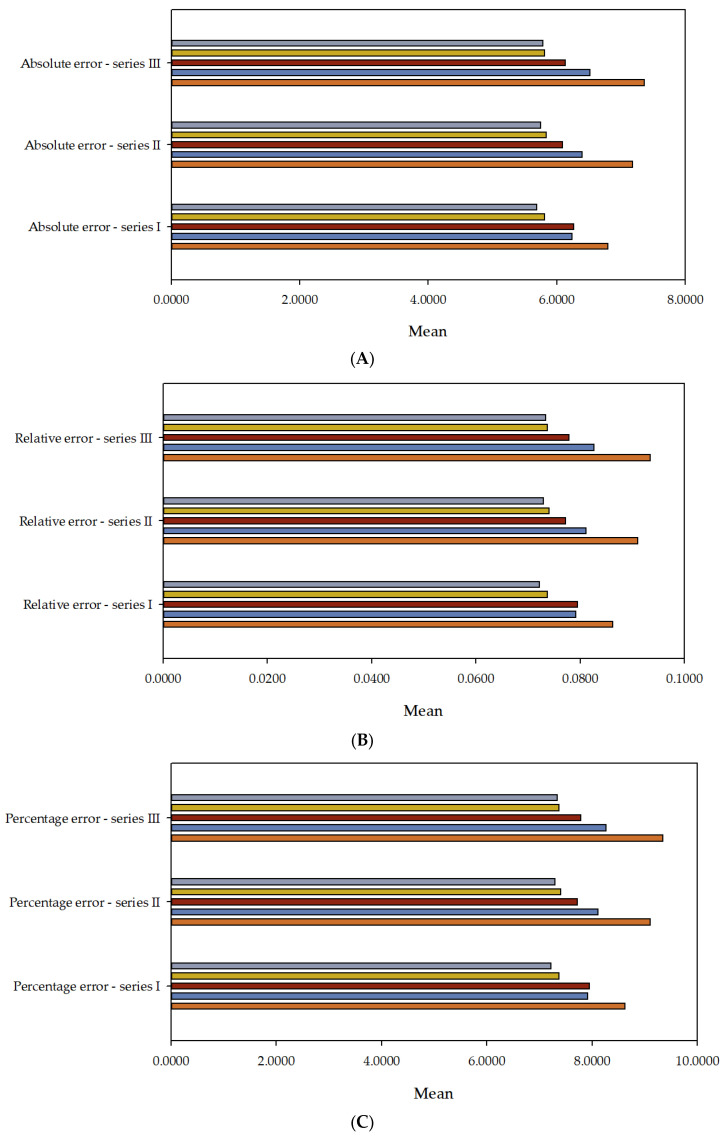
The values of mean error (absolute error (**A**), relative error (**B**), percentage error (**C**)) for MLM1 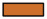, MLM2 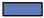, MLM3 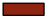, MLM4 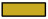, MLM5 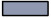 in series I, II, and III.

In Figure 11, a clear error downward trend can be noticed for the NSE metric in the training, selection, and testing sections and for the ME metric in the training section in all three series, with the increase in computational complexity. Simultaneously, a milder error downward trend can be observed in the remaining metrics, along with the increase in computational complexity. Statistical calculations were conducted for individual target variables, presented in Table 4. Table 4 provides minimums, maximums, averages, and standard deviations of absolute and percentage errors of the model for the test data. Figure 12 shows the values of mean error (absolute error, relative error, percentage error) for every model in series I, II, and III.

**Figure 13:**
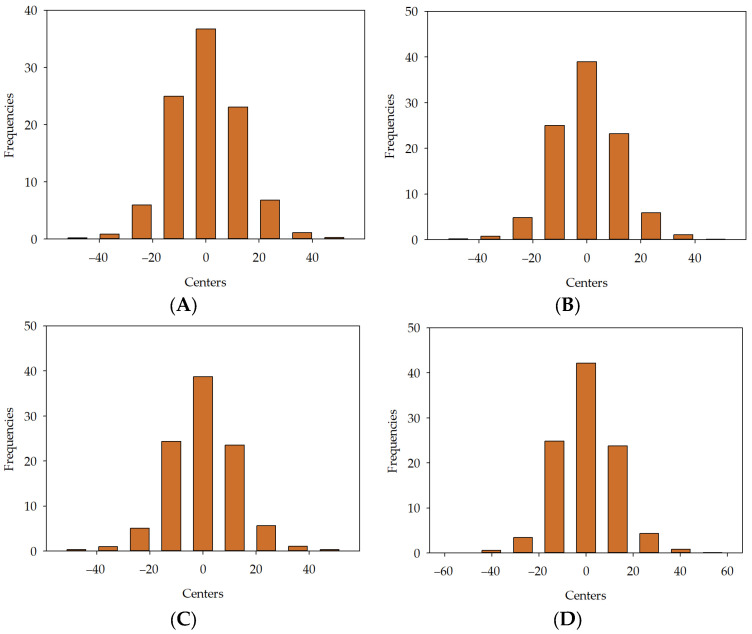

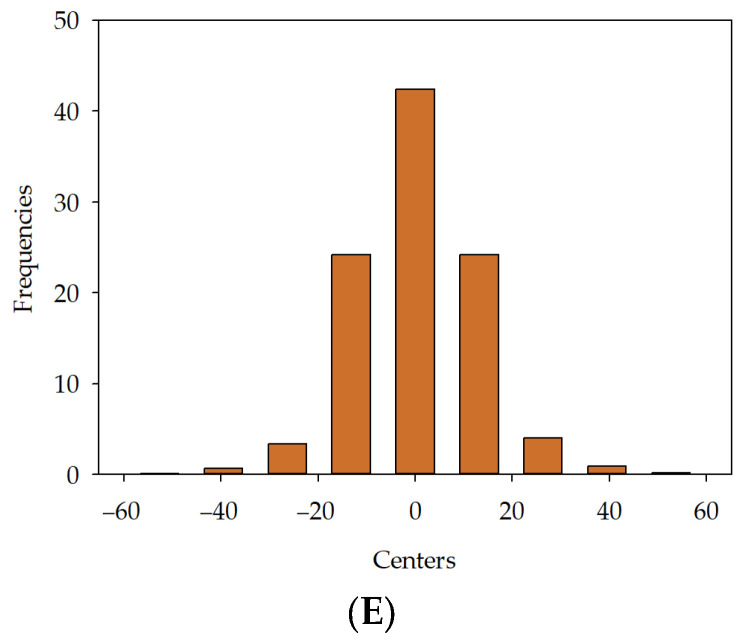
Error histogram for developed models in series I: MLM1 (**A**), MLM2 (**B**), MLM3 (**C**), MLM4 (**D**), MLM5 (**E**).

**Figure 14:**
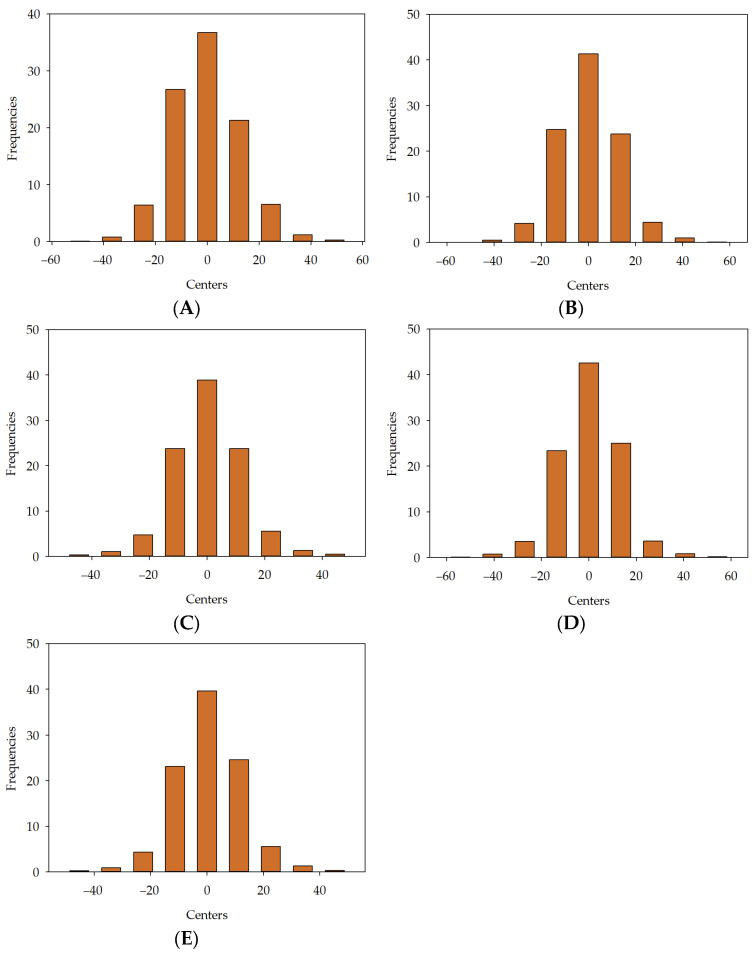
Error histogram for developed models in series II: MLM1 (**A**), MLM2 (**B**), MLM3 (**C**), MLM4 (**D**), MLM5 (**E**).

**Figure 15:**
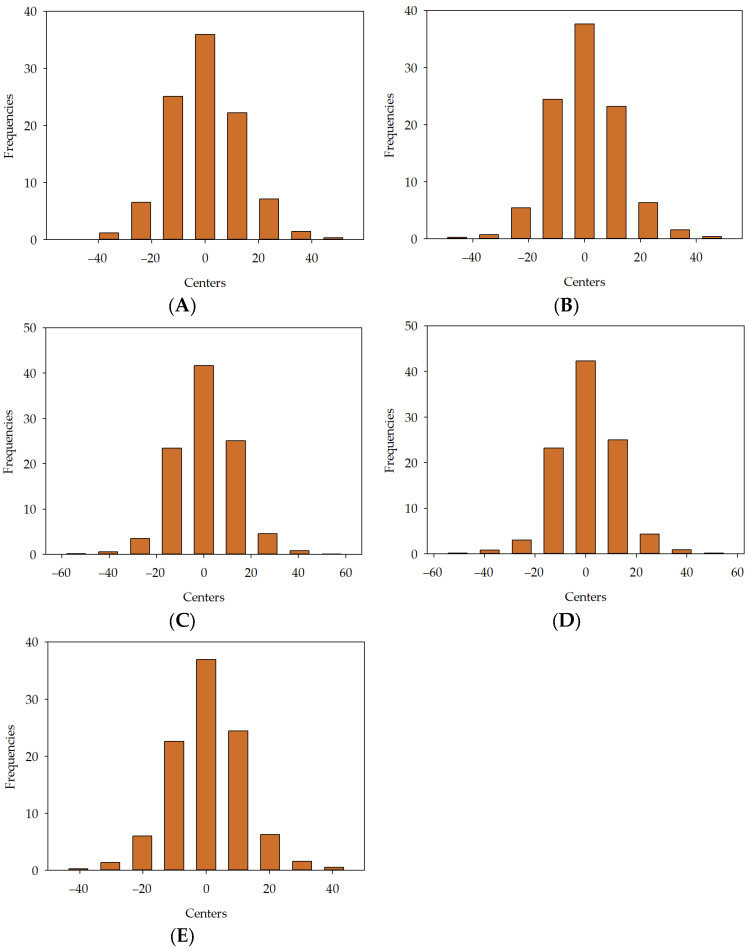
Error histogram for developed models in series III: MLM1 (**A**), MLM2 (**B**), MLM3 (**C**), MLM4 (**D**), MLM5 (**E**).

Analysing Table 4 and Figure 12A–C, one can observe that the average error decreases with the increase in the model’s computational complexity for absolute error, relative error, and percentage error. Figure 13, Figure 14 and Figure 15 represent the distribution of the relative error for the target variable f_ck_. The abscissa represents the centres of the containers, and the ordinate represents their corresponding frequencies. Error histograms show the distribution of errors from the model for the test subset. A normal distribution for the target variable is expected. For Figure 13A, the maximum frequency is 36.75%, which corresponds to the bin centred at 0%. The minimum frequency is 0.2%, which corresponds to the bins with centres at −48.277%. For Figure 13B, the maximum frequency is 39%, corresponding to the bin centred at 0%. The minimum frequency is 0.12%, corresponding to the bins with centres at −47.614%. For Figure 13C, the maximum frequency is 38.76%, which corresponds to the bin centred at 0%. The minimum frequency is 0.28%, which corresponds to the bins with centres at 47.656%. For Figure 13D, the maximum frequency is 42.11%, corresponding to the bin centred at 0%. The minimum frequency is 0.04%, corresponding to the bins with centres at −53.646%. For Figure 13E, the maximum frequency is 42.37%, which corresponds to the bin centred at 0%. The minimum frequency is 0.12%, which corresponds to the bins with centres at −52.676%.

For Figure 14A, the maximum frequency is 36.75%, which corresponds to the bin centred at 0%. The minimum frequency is 0.08%, which corresponds to the bins with centres at −49.165%. For Figure 14B, the maximum frequency is 41.3%, corresponding to the bin centred at 0%. The minimum frequency is 0.04%, corresponding to the bins with centres at −54.49%. For Figure 14C, the maximum frequency is 38.93%, which corresponds to the bin centred at 0%. The minimum frequency is 0.33%, which corresponds to the bins with centres at −44.438%. For Figure 14D, the maximum frequency is 42.6%, corresponding to the bin centred at 0%. The minimum frequency is 0.12%, corresponding to the bins with centres at −54.26%. For Figure 14E, the maximum frequency is 39.65%, which corresponds to the bin centred at 0%. The minimum frequency is 0.24%, which corresponds to the bins with centres at −45.284%.

For Figure 15A, the maximum frequency is 35.93%, which corresponds to the bin centred at 0%. The minimum frequency is 0.04%, which corresponds to the bins with centres at −48.049%. For Figure 15B, the maximum frequency is 37.62%, corresponding to the bin centred at 0%. The minimum frequency is 0.24%, corresponding to the bins with centres at −45.596%. For Figure 15C, the maximum frequency is 41.64%, which corresponds to the bin centred at 0%. The minimum frequency is 0.12%, which corresponds to the bins with centres at −54.004%. For Figure 15D, the maximum frequency is 42.29%, corresponding to the bin centred at 0%. The minimum frequency is 0.2%, corresponding to the bins with centres at −50.63%. For Figure 15E, the maximum frequency is 36.95%, which corresponds to the bin centred at 0%. The minimum frequency is 0.24%, which corresponds to the bins with centres at −40.473%.

It should be noted that all error histograms presented in Figure 13, Figure 14 and Figure 15 have a bell shape, which indicates a correctly obtained normal distribution [108,109]. The models are designed to estimate concrete compressive strength as determined by the composition of the concrete mixture. It is important to bear in mind that numerous additional elements, primarily linked to the technological process of concrete production and environmental conditions, exert influence over the concrete’s strength. The initial critical aspect involves the proper maintenance of concrete after the setting process. Incorrect handling of concrete can lead to a substantial deterioration its attributes, particularly its durability. Taking environmental aspects into account, it is essential to consider the effect of environmental aggression [110] that detrimentally affect concrete quality, such as frost action or exposure large amounts of alkalis. One of pivotal concern is pouring concrete under unfavourable weather conditions, particularly subjecting it to excessive shrinkage due to rapid drying under high temperatures or freezing early in the setting process. The arrangement and size distribution of the aggregates dictate the requirements for an appropriately workable concrete paste as well as the source, shape, and texture of aggregates. It impact especially the workability and durability of the concrete [111,112]. Admixtures and additives are also important in whole process, especially those that profoundly affect the chemical properties of the mixture. This assessment has omitted several factors, including environmental elements, technological processes, and properties of raw materials. It is assumed that the quality of the produced concrete samples met acceptable standards. The source code of all AI models presented in this research is available in an open repository [113].

## 4. Summary and Conclusions

In concrete structure production technology, a key challenge lies in ensuring predictable characteristics in the raw concrete mix and the hardened end product. Concrete mix manufacturers are responsible for guaranteeing that the concrete they deliver to construction sites meets the desired standards. However, achieving these standards consistently throughout the manufacturing process can prove daunting. Reliable prediction of concrete technical parameters is intricate, and most current solutions in the engineering field are estimations, which have increasingly become obsolete due to rapid advancements in material engineering. Predictive analytics dedicated to forecasting various phenomena, attributes, and patterns based on machine learning holds the potential to enhance the methodology behind concrete mix design substantially. The paper analyses the impact of computational complexity on the effectiveness of predicting the compressive strength of concrete using machine learning models. This study focuses on the growing interest in applying machine learning algorithms in material engineering, specifically in predicting the compressive strength of concrete, a key indicator of its quality. Computational complexity in this research refers to the number of hidden layers in the neural network architecture. In the context of machine learning models, computational complexity is essential, as it can influence the speed and effectiveness of the model’s training and its ability to generalise to new data. This study evaluated five deep neural network models (MLM1, MLM2, MLM3, MLM4, MLM5) of varying computational complexity in three series. Each of the MLM1-MLM5 models underwent training, selection, and testing in each three series. The crux of this research was to establish an ideal deep neural network structure and train it using a vast database of concrete mix recipes and their associated laboratory-based destructive tests.

Presented machine learning models predicts the compressive strength of the concrete mix based on its unique composition. Based on the obtained results, the following conclusions can be formulated. There is a relationship between the computational complexity of deep neural network models and their ability to predict the compressive strength of concrete. The conducted analyses showed that as the computational complexity of the model increases, so does its predictive ability. This means that the more complex the neural network architecture, the more effective it is in predicting the compressive strength of concrete within the conducted analyses. Several parameters point to the above conclusion. In all three series, the coefficient of determination (R^2^) increase was observed along with the increase in the model’s computational complexity. The smallest value of R^2^ was captured for model MLM1 and the largest for MLM5. It can be observed that errors in the five analysed metrics, namely mean squared error (MSE), Minkowski error (ME), normalized squared error (NSE), root mean squared error (RMSE), sum squared error (SSE), in training, selection, and testing decrease with the increase in the model’s computational complexity, with the greatest error decrease observable for the NSE metric in training, selection, and testing and the ME metric in training. Furthermore, it is worth examining the mean values of absolute error, relative error, and percentage error, which tend to decrease as the computational complexity of the model increases. The error histograms for all analysed models in all series have a normal distribution. While the proposed method offers a promising solution, it possesses certain limitations and does not comprehensively encapsulate all the interactions between concrete mix components and their resulting properties. This is a research gap that necessitates further exploration. Nonetheless, the outcomes of this paper inspire optimism for this method’s expanded application in practical engineering settings.

Future investigations should aim to broaden this method’s utility in the concrete mix design process by predicting additional fresh and hardened concrete properties such as durability, workability, air entrainment, and reliability. A more holistic strategy for optimising concrete mix design is also essential to development. The findings from this research lay a robust groundwork for further refinement and application of the proposed AI-driven method in concrete mix design. AI-driven methods automate the traditionally labour-intensive tasks, granting civil and structural engineers the liberty to tackle more intricate and inventive challenges. It is anticipated that this method will improve both the efficiency and precision of the design process, particularly in an era where advancements in hardware are accelerating the computational capabilities of AI.

## Figures and Tables

**Table 1:** The parameters utilized within the dataset.

Parameter	Concrete Compressive Strength	Cement	Water–Cement Ratio	Fine Aggregate	Coarse Aggregate
Type	Target	Input	Input	Input	Input
Description	The 28-day compressive strength of concrete that is considered to have most of its strength (MPa).	Content of cement added to the mixture (kg/m^3^).	Water-to-cement ratio (-).	Content of fine aggregate added to the mixture (kg/m^3^).	Content of coarse aggregate added to the mixture (kg/m^3^).

**Table 2:** Ranges of input variables for the database.

Input Variable	Minimum	Maximum	Mean	Median	Dominant
Cement	87.00 kg/m^3^	540.00 kg/m^3^	322.15 kg/m^3^	312.45 kg/m^3^	380.00 kg/m^3^
Water–cement ratio	0.30	0.80	0.58	0.58	0.58
Fine aggregate	472.00 kg/m^3^	995.60 kg/m^3^	767.96 kg/m^3^	774.00 kg/m^3^	594.00 kg/m^3^
Coarse aggregate	687.80 kg/m^3^	1198.00 kg/m^3^	969.92 kg/m^3^	963.00 kg/m^3^	932.00 kg/m^3^

**Table 3:** Error metrics for various data subsets.

Series I			
MLM1			
	Training	Selection	Testing
Sum squared error	550.201	342.198	317.183
Mean squared error	0.149	0.278	0.258
Root mean squared error	0.386	0.528	0.508
Normalised squared error	0.416	0.479	0.432
Minkowski error	1893.670	975.049	911.882
MLM2			
	Training	Selection	Testing
Sum squared error	489.493	310.386	295.271
Mean squared error	0.133	0.253	0.240
Root mean squared error	0.364	0.503	0.490
Normalised squared error	0.330	0.394	0.374
Minkowski error	1680.550	876.432	842.034
MLM3			
	Training	Selection	Testing
Sum squared error	458.547	313.610	305.511
Mean squared error	0.124	0.255	0.248
Root mean squared error	0.353	0.505	0.498
Normalised squared error	0.289	0.402	0.400
Minkowski error	1561.140	865.786	860.635
MLM4			
	Training	Selection	Testing
Sum squared error	447.218	307.594	290.030
Mean squared error	0.121	0.250	0.236
Root mean squared error	0.348	0.500	0.485
Normalised squared error	0.275	0.387	0.361
Minkowski error	1510.870	842.743	807.330
MLM5			
	Training	Selection	Testing
Sum squared error	416.643	296.432	291.111
Mean squared error	0.113	0.241	0.236
Root mean squared error	0.336	0.491	0.486
Normalised squared error	0.239	0.359	0.364
Minkowski error	1403.290	810.031	798.990
Series II			
MLM1			
	Training	Selection	Testing
Sum squared error	559.448	340.780	325.536
Mean squared error	0.152	0.277	0.264
Root mean squared error	0.389	0.527	0.514
Normalised squared error	0.431	0.475	0.455
Minkowski error	1944.850	974.611	947.078
MLM2			
	Training	Selection	Testing
Sum squared error	493.298	322.494	305.668
Mean squared error	0.134	0.262	0.248
Root mean squared error	0.366	0.512	0.498
Normalised squared error	0.335	0.425	0.401
Minkowski error	1687.480	905.087	867.447
MLM3			
	Training	Selection	Testing
Sum squared error	454.568	310.576	297.960
Mean squared error	0.123	0.253	0.242
Root mean squared error	0.351	0.503	0.492
Normalised squared error	0.284	0.394	0.381
Minkowski error	1539.040	852.571	837.393
MLM4			
	Training	Selection	Testing
Sum squared error	423.191	298.929	297.827
Mean squared error	0.115	0.243	0.242
Root mean squared error	0.339	0.493	0.492
Normalised squared error	0.246	0.365	0.381
Minkowski error	1431.560	823.304	820.941
MLM5			
	Training	Selection	Testing
Sum squared error	435.508	304.092	286.750
Mean squared error	0.118	0.247	0.233
Root mean squared error	0.344	0.497	0.483
Normalised squared error	0.261	0.378	0.353
Minkowski error	1458.890	834.351	799.642
Series III			
MLM1			
	Training	Selection	Testing
Sum squared error	575.272	347.520	332.199
Mean squared error	0.156	0.283	0.270
Root mean squared error	0.395	0.532	0.519
Normalised squared error	0.455	0.494	0.473
Minkowski error	2007.800	1004.980	969.083
MLM2			
	Training	Selection	Testing
Sum squared error	500.105	321.972	307.902
Mean squared error	0.136	0.262	0.250
Root mean squared error	0.368	0.512	0.500
Normalised squared error	0.344	0.424	0.407
Minkowski error	1706.130	905.095	879.370
MLM3			
	Training	Selection	Testing
Sum squared error	473.081	318.669	301.983
Mean squared error	0.128	0.259	0.245
Root mean squared error	0.358	0.509	0.495
Normalised squared error	0.308	0.415	0.391
Minkowski error	1608.550	889.350	844.676
MLM4			
	Training	Selection	Testing
Sum squared error	419.356	315.651	295.121
Mean squared error	0.114	0.257	0.240
Root mean squared error	0.337	0.507	0.490
Normalised squared error	0.242	0.407	0.374
Minkowski error	1407.230	862.350	814.506
MLM5			
	Training	Selection	Testing
Sum squared error	455.535	317.058	283.630
Mean squared error	0.123	0.258	0.230
Root mean squared error	0.351	0.508	0.480
Normalised squared error	0.286	0.411	0.345
Minkowski error	1529.650	866.807	798.284

**Table 4:** Error statistics for calculation of target value f_ck_.

Series I				
	Minimum	Maximum	Mean	Deviation
Absolute error				
MLM1	0.00000119209	0.0695368	0.00936655	0.00818558
MLM2	0.00000104308	0.0120825	0.00145332	0.00118896
MLM3	0.000000119209	0.0879388	0.00273278	0.0041617
MLM4	0.00000119209	0.148637	0.00331478	0.00663424
MLM5	0.00000119209	0.148637	0.00331478	0.00663424
Relative error				
MLM1	0.0000011413	0.0665743	0.00896749	0.00783684
MLM2	0.00000851071	0.0985832	0.011858	0.009701
MLM3	0.000000296304	0.218579	0.00679255	0.0103442
MLM4	0.00000213266	0.265913	0.00593016	0.0118687
MLM5	0.00000119209	0.148637	0.00331478	0.00663424
Percentage error				
MLM1	0.00011413	6.65743	0.896749	0.783684
MLM2	0.000851071	9.85832	1.1858	0.9701
MLM3	0.0000296304	21.8579	0.679255	1.03442
MLM4	0.000213266	26.5913	0.593016	1.18687
MLM5	0.00000119209	0.148637	0.00331478	0.00663424
Series II				
	Minimum	Maximum	Mean	Deviation
Absolute error				
MLM1	0.00000119209	0.0695368	0.00936655	0.00818558
MLM2	0.00000104308	0.0120825	0.00145332	0.00118896
MLM3	0.000000119209	0.0879388	0.00273278	0.0041617
MLM4	0.00000119209	0.148637	0.00331478	0.00663424
MLM5	0.00000119209	0.148637	0.00331478	0.00663424
Relative error				
MLM1	0.0000011413	0.0665743	0.00896749	0.00783684
MLM2	0.00000851071	0.0985832	0.011858	0.009701
MLM3	0.000000296304	0.218579	0.00679255	0.0103442
MLM4	0.00000213266	0.265913	0.00593016	0.0118687
MLM5	0.00000119209	0.148637	0.00331478	0.00663424
Percentage error				
MLM1	0.00011413	6.65743	0.896749	0.783684
MLM2	0.000851071	9.85832	1.1858	0.9701
MLM3	0.0000296304	21.8579	0.679255	1.03442
MLM4	0.000213266	26.5913	0.593016	1.18687
MLM5	0.00000119209	0.148637	0.00331478	0.00663424
Series III				
	Minimum	Maximum	Mean	Deviation
Absolute error				
MLM1	0.00000119209	0.0695368	0.00936655	0.00818558
MLM2	0.00000104308	0.0120825	0.00145332	0.00118896
MLM3	0.000000119209	0.0879388	0.00273278	0.0041617
MLM4	0.00000119209	0.148637	0.00331478	0.00663424
MLM5	0.00000119209	0.148637	0.00331478	0.00663424
Relative error				
MLM1	0.0000011413	0.0665743	0.00896749	0.00783684
MLM2	0.00000851071	0.0985832	0.011858	0.009701
MLM3	0.000000296304	0.218579	0.00679255	0.0103442
MLM4	0.00000213266	0.265913	0.00593016	0.0118687
MLM5	0.00000119209	0.148637	0.00331478	0.00663424
Percentage error				
MLM1	0.00011413	6.65743	0.896749	0.783684
MLM2	0.000851071	9.85832	1.1858	0.9701
MLM3	0.0000296304	21.8579	0.679255	1.03442
MLM4	0.000213266	26.5913	0.593016	1.18687
MLM5	0.00000119209	0.148637	0.00331478	0.00663424

## Data Availability

Not applicable.
